# Urban intelligent assistant on the example of the escalator passenger safety management at the subway stations

**DOI:** 10.1038/s41598-023-42535-x

**Published:** 2023-09-23

**Authors:** Man Tianxing, Alexander Vodyaho, Nataly Zhukova, Alexey Subbotin, Yulia Shichkina

**Affiliations:** 1 School of Artificial Intelligence, Jilin University, Changchun, 130012 China; 2 Saint-Petersburg State Electrotechnical University “LETI”, St. Petersburg, 197022 Russia; 3 Laboratory of Big Data Technologies in Socio-Cyberphysical Systems, Saint-Petersburg Federal Research Centre of the Russian Academy of Sciences, St. Petersburg, 194021 Russia

**Keywords:** Computer science, Information technology

## Abstract

Intelligent assistants often struggle with the complexity of spatiotemporal models used for understanding objects and environments. The construction and usage of such models demand significant computational resources. This article introduces a novel multilevel spatiotemporal model and a computationally efficient construction method. To facilitate model construction on different levels, we employ a meta-mining technique. Furthermore, the proposed model is specifically designed to excel in foggy environments. As a practical application, we develop an intelligent assistant focused on enhancing subway passenger safety. We present case examples involving jammed objects, such as shoes, in escalator combs. Our results demonstrate the effectiveness of the proposed model and method. Specifically, the accuracy of breakdown detection has improved by 10% compared to existing information systems used in subways. Moreover, the time required to build a spatiotemporal model is reduced by 2.3 times, further highlighting the efficiency of our approach. Our research offers a promising solution for intelligent assistants dealing with complex spatiotemporal modeling, with practical applications in ensuring subway passenger safety.

## Introduction

With significant advancements in various fields of knowledge and the rapid progress of data mining (DM) and machine learning (ML) techniques, the creation of intelligent assistants has become feasible, leading to their widespread use in everyday life. These modern intelligent assistants effectively tackle a wide range of practical tasks in diverse domains, including smart homes and industries^1–3^. However, despite the high demand and their broad applications, intelligent assistants are still underutilized when it comes to monitoring and managing technically complex urban infrastructure elements, such as subway systems. In the context of subways, these assistants are expected to handle functions related to monitoring and management, based on the processing of real-life intricate data, including heterogeneous, spatiotemporal, and dynamic information. This data is collected from the observed objects using traditional monitoring systems.

The intelligent capabilities of assistants heavily rely on the models they utilize. However, the high computational complexity involved in building and rebuilding spatiotemporal models often leads to the application of static models, which are expert-constructed using machine learning techniques. Although various self-learning methods have been proposed to update these models, they do not allow for dynamic model rebuilding, which hinders keeping the models up-to-date. Consequently, this has led to the development of numerous specialized models tailored to specific tasks or limited task ranges. While specialized models have proven effective in practice, the increasing number of tasks makes this approach increasingly impractical.

To address this issue, constructing models with a multilevel structure enables data processing at different levels. In typical scenarios where objects exhibit regular behavior and no extraordinary events significantly influence their state, data processing occurs at the upper levels of the model. The number of elements at these upper levels is significantly smaller than that at the lower levels, resulting in reduced computational resources and time requirements. However, when extraordinary events occur or the behavior of objects unexpectedly changes, the model transitions to lower levels, allowing for detailed consideration of the objects' states and ongoing processes. This transition to lower levels empowers the assistant to achieve a higher level of accuracy in providing results.

The proposed method for multilevel model construction has successfully addressed the challenge of ensuring lower computational complexity compared to existing methods, enabling the construction of complex spatiotemporal models for intelligent assistants while facilitating dynamic model reconstruction. The new multilevel model for the intelligent assistant possesses the following distinguishing features:

- *Spatial models* allow track states of spatially distributed objects^4,5^;
- *Temporal models* allow consideration of events that are registered on the objects at different moments of time;
- *Dynamic models* are designed to reflect the current state of the observed objects; they are rebuilt with minimal delay after changes in the behaviour or states of the objects occur^6,7^ using operational data received from the objects.

Furthermore, constructing models with multilevel structures offers the advantage of visualizing data pertaining to the observed objects and the outcomes of data processing at different scales corresponding to the levels of the model. This visualization capability enhances the accessibility and user-friendliness of the data, enabling users to comprehend and analyze the information more effectively^8–10^.

In summary, the key contributions of this paper can be summarized as follows:

1. A new spatiotemporal model for building intelligent assistants whose construction and reconstruction have low computational complexity. The intelligent assistants use AI techniques for solving tasks of monitoring and management of technically complex social objects of urban infrastructure.
2. An intelligent assistant for subway passenger safety management deployed in the subway is successfully used by the operators in their daily work.
3. The results of the experiments show the efficiency of using intelligent assistants for the support of operators of technically complex social objects and demonstrate the usefulness of further development of the proposed AI-enabled intelligent assistants.

The second section of the paper presents an in-depth review of intelligent assistants, encompassing their classification, main functions, and capabilities. Moving to the third section, it articulates the central problem: the development of intelligent assistants tailored to tackle complex tasks, notably ensuring the safety of escalator passengers in subway environments. Sections “Meta-mining-based workflow construction” and “Methods” delve into the proposed solution, providing comprehensive details about the multilevel spatiotemporal model. The case study then showcases the implementation of the model in the subway, along with the results obtained from real-world usage. Finally, the paper culminates with a conclusive section that summarizes key findings and emphasizes the effectiveness of the proposed model for addressing intricate challenges in subway safety management.

## Background

Intelligent assistants are commonly regarded as software agents capable of performing tasks or providing services, typically through conversational interfaces. Various definitions exist in the literature^45^, with the majority emphasizing their high level of intelligence. These assistants are expected to recognize voices, comprehend questions, model human behavior, and even predict user needs. They achieve this by leveraging artificial intelligence based on neural networks and machine learning, enabling seamless communication across extensive networks comprising machines, computers, and distant individuals.

Currently, intelligent assistants are in high demand across diverse user groups^45^:

- housewives for obtaining kitchen recipes, weather forecasts, etc.;
- people with limited mobility^49^ and blind people^48^;
- travellers who need help in translating from foreign languages;

Assistants are also used for^51^ multiple situations:

- monitoring the states of technical systems (ventilation, air conditioning, automatic garage doors and other systems that are components of a "Smart Home");
- managing security systems in premises (residential buildings, factories, etc.);
- undergoing authorisation using voice to confirm the password (computer, car, checkpoint, etc.).

Intelligent assistants have emerged as vital components in the development of smart cities^48^, smart factories^58^, smart medicine^54^, etc. Though by now a number of AI-enabled assistants have been already proposed^58,62–64^, still further extensive research in the direction of increasing the level of their intelligence is highly required, in particular, there is a need in intelligent assistants that can use AI techniques for monitoring and management of technically complex objects and systems, including social objects of smart urban infrastructure^53^.

By now, several taxonomies have been developed that allow estimate the assistants from different perspectives^45–47^. Primarily, the assistants can be classified based on their design and the domain of application.

From the design perspective, intelligent assistants are classified as follows^11,12^:

- mobile applications that ensure recognition of voice, sounds, and images from smartphone sensors;
- stationary speakers with a microphone and internet connection;
- components of technologically embedded computers.

Presently, there is a plethora of markets offering a diverse array of applications. For instance, within the realm of cooking, multiple applications cater to various needs. The latest generation of smartphones extends beyond the provision of simple recipes; they are now equipped with advanced features that enable users to determine the degree of readiness of a dish through video camera and heat sensor integration. Moreover, emerging technologies are poised to introduce even more capabilities, such as capturing odours using microparticle traps and various other innovations^13,14^. In particular, the forthcoming developments of Apple for smartphones based on iOS are aiming to build such applications^15^.

Stationary speakers are applications with quite limited functionality that provide requested references from the internet and allow ordering goods, considering the user’s location and preferences. They also allow search for music to listen to, obtaining weather forecasts, conducting simple dialogues, and obtaining information from the address bar of the browser and from Wikipedia using voice. Currently, the most popular stationary speakers that are often referred to as assistants^16,17^ are Siri, Google Assistant, Amazon Alexa, Microsoft Cortana, Bixby, Voice Mate, Alice, Marusya, Dusya, and Salut. These types of systems include software for TVs, projectors, and voice-controlled multimedia centres^18,19^. The majority of them are not always autonomous and cannot work without a PC.

Technological computers are frequently integrated as essential components within intricate technical systems. For instance, an intelligent assistant incorporated into a ship's technological computer is responsible for overseeing various critical functions, such as controlling the ventilation system, managing energy consumption, ensuring the security system's operation, and facilitating seamless communication and interaction with coastal services and navigation systems^20^.

From the view of the application domain, the following main groups of assistants can be distinguished: the assistants that provide general functions for retrieving information, searching on the web or playing music and specific assistants developed for solving certain complex tasks or developed for the dedicated user group^46^.

To general assistants refer intelligent assistants for modern smart homes, where they enable lighting level regulations and energy management. Assistants can change the heat level by controlling the heating system and the coolant supply^21^ using smart gas boilers that can be automatically turned on and off via SMS. A smart assistant can also gather information about the environment and regulate the production of electricity using the bowels of the Earth, such as solar, wind energy and other natural energy sources that allow generate electricity for the smart home^22–24^. Also, assistants have been developed for car drivers^57^, for business^56^, for shopping^60^ as well as many others.

**Figure 1:**
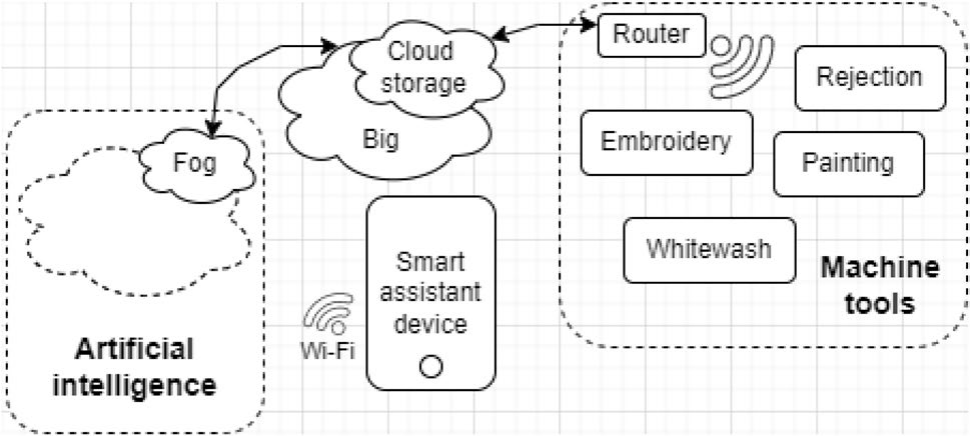
Intelligent assistant of a factory.

Specific assistants have been developed for a number of subject domains including industry, medicine, education, sport^47,50,51,53,54,59^. For example, in the industry domain intelligent assistants are used in factories for fabric production (Fig. 1). In particular, the controller that checks the quality of the products uses intelligent assistants endowed with image recognisability^25,26^. Additionally, assistants allow recognition of the sounds of machine malfunctions to prevent future tragedies. In dangerous productions, such as the production of bricks and tiles, applying the assistants ensures temperature and technological control. At all stages of production, intelligent assistants analyse sounds and video information including images from smartphone cameras and images gathered centrally from video cameras deployed in the factory to obtain more accurate results^27–29^. In addition to sensors and manipulators, smart rings are used to record the behaviour and downtime of employees. However, as the results of the experiments show, smart rings with tracking functions have a negative impact: people quit and do not work as well since any specialist needs a certain degree of freedom.

To date, intelligent assistants, regardless of where they are used are expected to be complex systems that implement extended functions for managing complicated processes, including monitoring, interaction with third-party services^30–32^, etc. and can be easily used both in daily life and at work.

The assessment of intelligent assistants' capabilities relies on their defining characteristics, which primarily revolve around their level of interaction and intelligence^46,55^ and by the technical means that they require. The characteristics that specify the degree of interaction refer the communication mode (text/voice/vision), type of communication (natural language/formal language), direction of explicit interaction (user-to-system interaction/system-to-user interaction/bidirectional interaction). The intelligence of the assistants is defined based on the provided functions, the complexity of the commands that it can execute, its adaptivity and embodiment.

Recent research has led to significant advancements in modern intelligent assistants, particularly in the realm of voice interaction with users. These advancements have been achieved through the implementation of extended models and sophisticated methods for natural language processing^52,61^. Specialized assistants, such as Viv, have been developed, enabling comprehension of complex user queries. However, one prevailing limitation is that many assistants are confined to a single language, be it Russian, English, Chinese, and so on. For instance, Yandex's Alisa and Mail.ru Group's Marusya are examples of assistants predominantly operating in one language. Moreover, the voices of these assistants are challenging to modulate, often relying on real voices. While slight modifications within the verbal set and individual sound pairs are possible, altering the timbre of the voice can reveal the artificial nature of the assistant, hampering the user experience. Furthermore, a notable observation is that only a few assistants provide users with easily interpretable visual representations of information during interactions. Specifically, most assistants lack the capability to construct navigable 2D/3D models, which are crucial for tasks involving object monitoring and management.

From a functional perspective, the considered assistants exhibit key limitations, as each is capable of addressing only distinct specialized tasks that involve utilizing maps, information from the internet, and various social institutions and services. For example, some assistants excel in route planning (e.g., Ozlo), while others excel in searching within text messengers of social networks (e.g., Facebook M, Xiao Ai). Additionally, specific assistants are adept at tasks such as movie searching, music recognition (e.g., SoundHound), travel guidance (e.g., Robin), providing background information, food ordering, playing games (e.g., Microsoft Cortana), and controlling operating systems (e.g., Windows Typle). Furthermore, several assistants have additional limitations. For instance, certain music assistants are restricted to working only with specific music providers (e.g., Apple Music, Google Play Music, Okko, VK Music). Similarly, assistants facilitating food ordering are limited to supporting only a select number of food vendors (e.g., Domino's and Pizza Hut). The primary cause of these limitations stems from the fact that the functions provided by current assistants are based on executing predefined actions or simple sequences of actions. As a result, almost no assistants possess the capability to dynamically construct and reconstruct complex processes that enable the resolution of non-formalized tasks in a changing environment. Such tasks, for instance, pertain to the monitoring and management of technically complex objects, necessitating the utilization of AI techniques. Addressing these limitations calls for further research and development in intelligent assistants, aiming to imbue them with dynamic adaptability and the capacity to solve complex, real-world tasks in a flexible and intelligent manner. This evolution will undoubtedly contribute to enhancing the overall effectiveness and utility of intelligent assistants in diverse applications and domains.

Many intelligent assistants are developed exclusively for specific operating systems, such as Mac, iOS, WatchOS, Windows, Android, FireOS, and XboxOS. Additionally, some assistants are designed to work solely with certain brands or utilize specific technologies, such as Apple, Google, or Microsoft. This hardware and software dependency significantly limits the widespread use of these assistants, as not all users possess devices that meet the required characteristics.

Upon analyzing the existing assistants, it becomes evident that their capabilities are rather limited, rendering them inadequate as specialized assistants for addressing applied tasks related to the monitoring and management of technically complex objects and systems. These tasks are in high demand across various domains, including smart cities, smart medicine, and smart industries. The key limitations observed in existing assistants are as follows:

- *Limited diagnostic information* Existing assistants can only gather diagnostic information about managed objects, which may no longer suffice for complex entities such as factories and plants. Specialized assistants are needed to not only collect data but also plan and manage processes, employee behavior, working hours, interactions with suppliers and consumers, as well as the operation of various components and mechanisms within complex technical systems.
- *Visual representation gap* While the majority of assistants utilize text and voice-based communication with users, specific assistants aimed at complex tasks should also provide advanced visual representation in the form of navigable 2D/3D models. This capability is crucial for enhanced understanding and decision-making in complex scenarios.
- *Hardware and platform dependency* Many existing assistants are constrained by their reliance on specific hardware, platforms, or software, necessitating users to purchase specialized devices to access their functionalities.

Indeed, the current landscape of intelligent assistants highlights a significant gap in the field. Specifically, there is a lack of a specialized AI-enabled assistant capable of addressing the tasks associated with monitoring and managing technically complex social objects. The need is for an assistant with high intelligence, proficient in constructing and dynamically adjusting complex processes using data collected by monitoring systems equipped with sensors, video cameras, microphones, and other devices. Such an assistant should seamlessly integrate with existing technical infrastructure, including technological embedded computers, fog servers, and smartphones, and be compatible with the software components of existing information systems. Furthermore, the assistant should interact with complex existing systems and collaborate with intelligent video surveillance operators to ensure efficient operation of managed objects, relying on well-founded solutions achieved through extensive utilization of artificial intelligence techniques.

To overcome the enumerated limitations and achieve the desired capabilities, the assistant requires a model of the monitored objects and systems. The newly developed theory of multilevel synthesis offers a promising solution, reducing the complexity and time required for model building and reconstruction significantly. This approach ensures the practical implementation of the assistant, enabling it to build complex processes for solving applied tasks in dynamic environments and providing information representation through 2D/3D models of the objects. Additionally, to foster widespread adoption, the use of platform-independent freeware program components during the design phase is proposed, ensuring versatility and accessibility.

The research paper aims to demonstrate the efficiency of the proposed intelligent assistant through an exemplary case study focused on ensuring the safety of escalator passengers in the subway. By showcasing the successful application of the assistant in this specific scenario, it contributes to bridging the gap in the field and opens up possibilities for future advancements in intelligent assistants for diverse applications in various domains.

## Problem definition

A complex production system based on IIoT stack is required to ensure the safety of passengers in the subway. However, ready-made systems meeting these specifications are not readily available, and existing developments are not tailored to the specific needs of the subway environment. In the envisioned system, the functions of information transfer would be assigned to smartphone applications. For the situation center operator, two applications need to be developed: a desktop application and a website with an adaptive layout design that works seamlessly across all types of devices. The processes within the production system should be managed by an intelligent assistant.

This intelligent assistant should be based on a multilevel spatiotemporal model, capable of resolving the challenges of place/event matching, which is crucial for identifying dangerous situations and visualizing events for operators' applications. The model serves as a digital representation of the subway, depicting it as a complex technical object. At the first level of the model, the state of the subway station at different time intervals is described. The second level provides details about individual elements of the station, including the lobby, platform, trains, passengers, escalators, and more. The third level offers detailed descriptions of specific elements like escalators, along with events that occur on them, such as objects getting stuck in the escalator combs, and enumerates the reasons for these events, such as untied shoelaces. The spatiotemporal properties of the model enable the construction of a timeline, facilitating time-travel to review past states of the station.

The constructed spatiotemporal model should be dynamic, representing real-time changes at the subway stations or with minimal delays. The model construction methods should facilitate easy building and rebuilding based on raw data gathered from sensors, video cameras, microphones, and other devices installed in the station. Additionally, existing models can be adapted using the gathered data and the results of processing.

**Figure 2:**
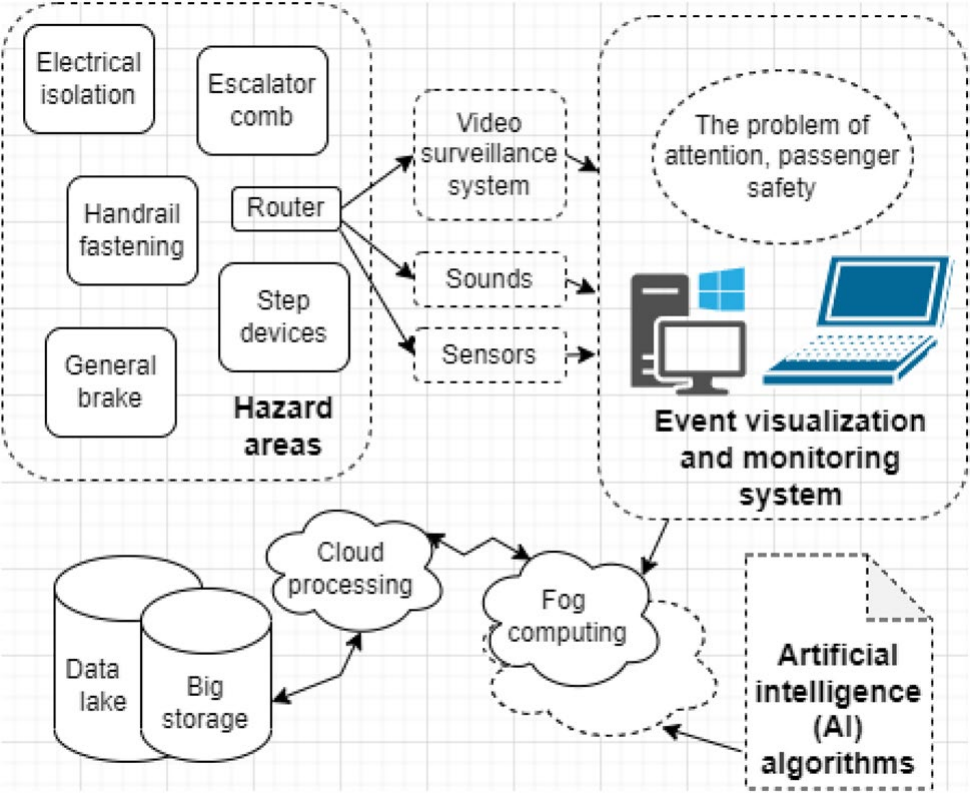
The problem of the attention and safety of passengers.

The systems for monitoring, event detection, and visualization of subway stations based on the multilevel spatiotemporal model require a new architecture (Fig. 2).

They should be deployed both in the fog computing environment and the cloud. Data collected from various devices, such as video streams, sounds, sensor data, and information from support specialists, is sent to the fog environment for processing using neural networks and machine learning algorithms^25^. The visualization of detected events should be provided through applications developed for desktop computers, websites, and smartphones, utilizing the information processed in the fog computing environment. Detected events and dangerous areas should be displayed on the screen, along with the observed objects they pertain to. For constant data storage, a combination of a database and data lake (server file system) in the cloud should be employed.

### Rationale for development

Each year, tragic incidents occur on the subway's underground railways, resulting in fatalities and a significant number of injuries, some of which lead to severe disabilities. Passengers frequently sustain injuries to their arms, legs, head, and other body parts with varying degrees of severity. These unfortunate events can be caused by fatal electric shocks, falls from escalator steps onto railway tracks or granite steps, entrapment in train doors and horizontal elevator doors, or injuries resulting from sudden train braking. Additionally, acts of hooliganism are not uncommon in the subway environment. It is necessary to reduce the number of injuries of both the employees of the subway and the passengers and to increase the effectiveness of prevention of dangerous situations at the stations and on the subway’s territory as a whole. This can be reached by developing a new system for the subway that will be able to ensure timely warning and prevention of dangerous situations due to using the proposed multilevel spatiotemporal model.

### Building a model

The implementation of the spatiotemporal model is expected to play a crucial role in timely identifying events that may lead to hazardous situations at subway underground stations, escalator combs, and other elements of the subway infrastructure. By utilizing this model, the new subway system can efficiently facilitate operators' interaction with technical systems and promptly provide assistance to passengers, ultimately leading to an overall improvement in the quality of service and safety within the subway environment.

## Meta-mining-based workflow construction

Since the data that is gathered in the subway is multimodal, spatial and dynamic, the proposed intelligent assistant should ensure building complex workflows for data processing that involve a wide application of machine learning techniques (ML workflows). ML workflow construction assumes building processes with hierarchical structures using meta-mining techniques that allow the selection of algorithms and models to process gathered data^33^ using knowledge bases presented in the form of ontologies that clarify the complex relationships between tasks, data, and algorithms at different stages in the ML process.

### DM process ontology

The DM process ontology as the knowledge base for building DM processes is based on the open standard process model CRISP-DM^34^. The ontology allows searching for suitable processes and algorithms according to the task requirements and the gathered data characteristics and defining the sequence of their application.

**Figure 3:**
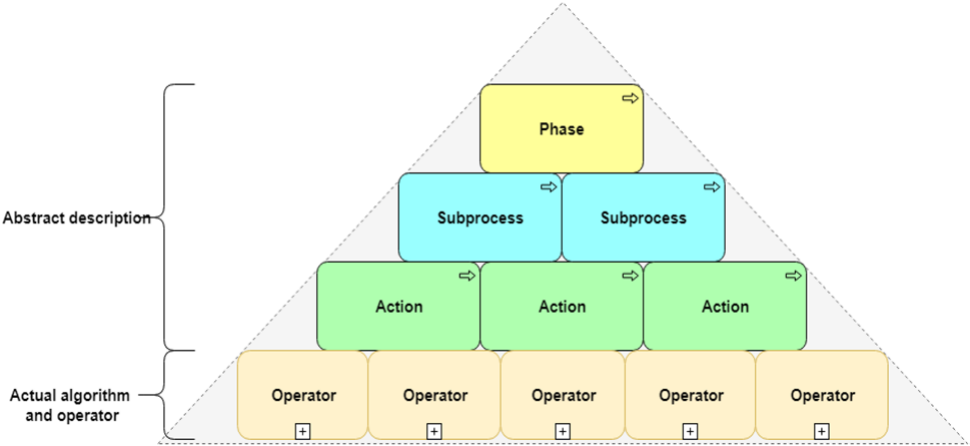
The hierarchical structure of the DM process ontology.

The proposed DM process ontology has a hierarchical structure at the top of which are abstract processes that are detailed at lower levels (Fig. 3). Four layers are defined: Phase, Subprocess, Action and Operator. The first three layers describe the abstract processes, and the last layer presents operators and algorithms^35^ that have software implementation. The proposed ontology integrates the OntoDM^36^, DMOP^37^ and OntoKDD^38^ ontologies, which provide the entities of the DM process, and OntoDT^39^, IAO^40^ and DMWF^41^, which provide the concrete operators, input and output objects.

**Figure 4:**
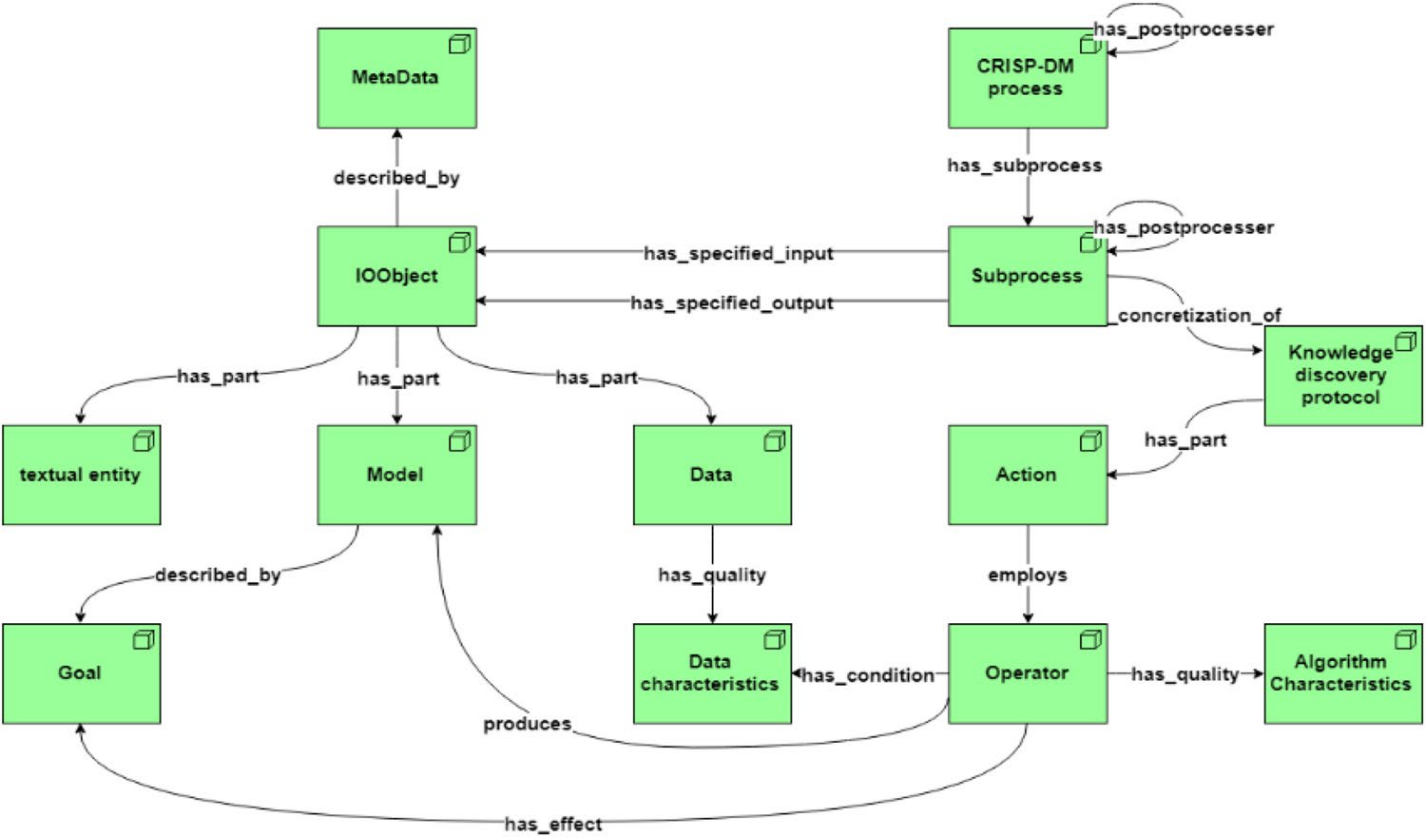
The hierarchical DM process ontology.

The main classes of the DM hierarchical process ontology are presented in Fig. 4.

### Rule-based interactive interface

The proposed DM ontology provides a framework for supporting DM workflow construction. Building workflows assumes constructing queries to ontologies using description logic (DL)^42^. The constructed queries are further applied to query the ontologies. For the cases when it is necessary to construct workflows manually, a rule-based interactive interface that supports query generation is provided. This type of interface allows queries to be constructed to specialists in subject domains, the majority of whom are noncomputer researchers. The proposed rule-based interface is built on the basis of Drools^43^.

**Figure 5:**
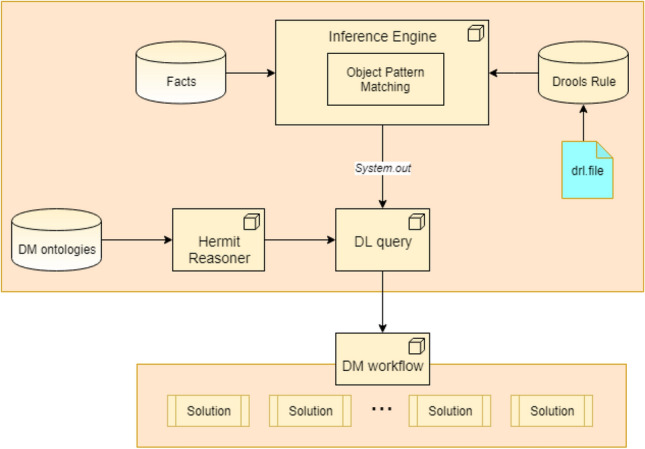
The framework of the rule-based interactive interface.

As shown in Fig. 5, the core of the Drools suite is an advanced inference engine that uses an improved Rete algorithm^44^ for object pattern matching. Rules are stored in the production memory, while facts are maintained in the working memory. The production memory remains unchanged during an analysis session, i.e., no rules are added, removed, or changed, but the contents of working memory can change. Facts may be modified, removed or added as a result of executing rules or when new facts are received from external sources. After a change in the working memory, the inference engine is triggered, and it determines which rules become “true” for the given facts.

In the proposed rule-based interface, the users’ requests define the input facts, and the necessary DL queries are output. The queries are generated using preset rules. The output DL queries are formatted as rules with two parts: 1. The fixed content шы presented in the form of a “String”; 2. The variables are presented as the names of the class properties.

### Multilevel workflow construction

The implementation of the construction of DM workflows with hierarchical structure is based on defining specific queries to the DM process ontology when constructing each layer. The queries are generated based on the designed Drools rules.

The construction of the DM workflows is performed on the following levels:

- The "Phase" layer is used to define the general sequence of the steps of the DM process. It follows the CRISP-DM framework and defines six phases. The sequence of the phases is specified by the property called "hasPostprocess”.
- The “Subprocess” layer is used to decide whether a step of the process is necessary for solving the specified task. Each subprocess has a specific input and output that are described using the class "IOObject"; for example, a precondition of the subprocess “handling_missing_attribute_values” is “has_specific_input some attributeWithMissingValues"; “attributeWithMissingValues” is a characteristic of the dataset that is described in “IOObject”.
- "Action" layer is used to define a list of actions that allow the implementation of the subprocesses defined at the ‘Subprocess’ layer and that are suitable for the data with the defined characteristics and defects. The relations between the entity action and data characteristics are defined by the property "suitableFor."
- The "Operator" layer is similar to the "Action" layer. However, it allows us to decide which algorithms are suitable for data with the defined characteristics and defects. The processes defined at the “Action” layer are the abstract processes, but for the processes at the “Operator” layer, the specific algorithms or operators are determined; for example, for the subprocess, “Imputation” the algorithm “KNN_Imputation_ED (K Nearest Neighbour algorithm with Euclidean distance)” should be used.

The inputs are the DL queries. The DL queries are used to query the DM ontologies. The result is the list of ontology entities or separate entities.

**Figure 6:**
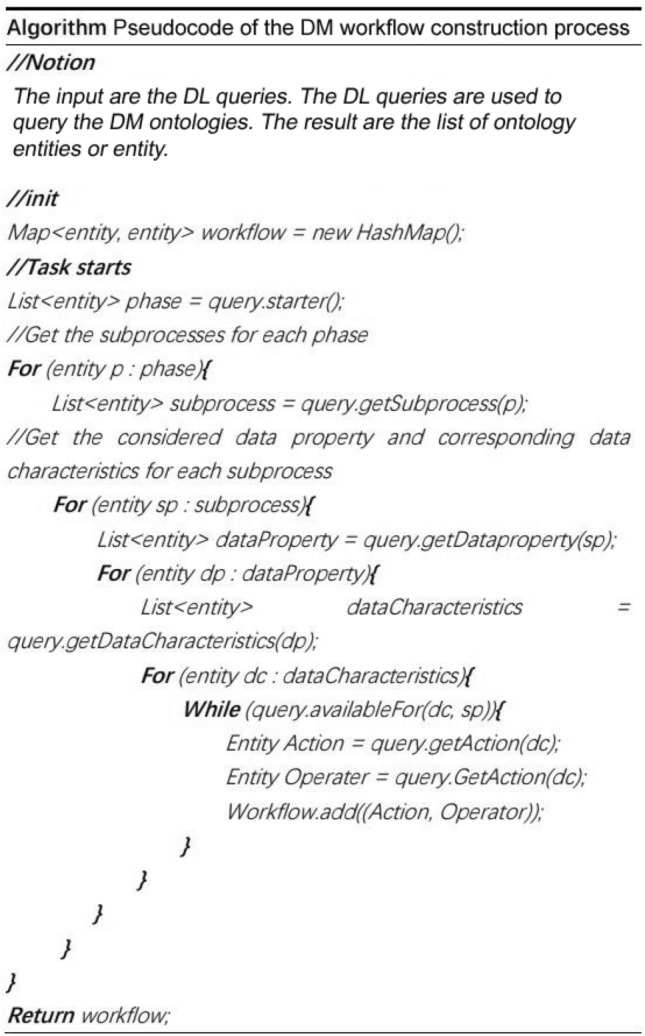
The pseudocode of the DM workflow construction process.

The pseudocode of the DM workflow construction process is presented in Fig. 6. In the case of manual workflow construction, the users employ the query module. They input the requests in the Drools program, obtain the DL query, and then run the DL query on the DM ontologies. The query results are a list of ontology entities or an entity.

## Methods

The multilevel models can be likened to digital twins of the subway stations, offering a comprehensive representation of the stations' characteristics. They serve as the foundation for constructing graphic 2D/3D models that accurately depict dangerous areas and past events that occurred at the stations. The models possess detailed information about events on the lower levels while providing an overview of the station's complex state on the upper levels. These models are continually updated to reflect the ever-changing dynamics at the subway stations. Thanks to the spatiotemporal properties of the models, events can be reviewed retrospectively, enabling a detailed examination of their progression over time. For instance, operators can revisit events that occurred 10, 20, 30 min, or 2 h ago to gain a comprehensive understanding of the unfolding situation. The models of different stations have much in common, but at the same time, each model considers the peculiar features of the station for which it is built.

The process of model building in the proposed system includes the following main steps:

1. Loading the program into RAM (the previously built model of the subway station is loaded);
2. Reading settings from files: conf_model.ini, setg_view.ini (screen sizes, video camera parameters: IP-addresses, logins, passwords, ports, compression modes, frames rate per second, colorizes, dimensions; description of drivers for sensors, microphones and other devices);
3. Checking the connections with all the devices (cameras, microphones, sensors), and diagnostic information is collected only once at the system boot;
4. Obtaining information in the form of a series of frames per second, an audio clips of three seconds long (± 1 s), data from discrete sensors (binary values) per frame second, and data from analogue sensors, which is processed in the same way as the data from the microphones. The system is configured in such a way that its parameters are the following: + 15% processor performance, + 10% RAM and + 25% network load (for the cases of force majeure);
5. Data processing using machine learning (ML) techniques. Three streams of obtained information are processed. The purpose of processing is to detect the events that have already occurred, to reveal precursors of future events and to estimate their probability. When the defined threshold of the probability is exceeded, the program notifies the operators;
6. Detected event identification; if no events have been detected, move to step 4. If the events have been detected, then the results of their identification are estimated; for example, 95% accuracy that the event is the fire;
7. Displaying events to the operators, in particular, intelligent video surveillance operators via a smartphone. On the smartphone, a user sees a digital twin of the subway station that is a copy of a subway station presented in the form of 2D or 3D models that are built using graphic primitives, such as points, straight lines and curves. It is possible to rotate the 3D models in space using cursor or mouse arrows. The representation of the 2D models is similar to the representation of images, and it is possible to move and scale the image, particularly to zoom in (Crtl+) and to zoom out (Ctrl−). The user can switch between 2 and 3D models. On the model, the events are visualised in the form of marker points. To draw the operator’s attention to marker points, red, yellow and blue colours are used depending on the degree of danger of the identified events. The digital twins in the smartphones are loaded once and then updated each time when the model of the subway station in the fog changes; for example, it was rebuilt based on the results of the processing of new data received from video cameras, smartphones and sensors.
8. Waiting for a reaction from the operator;
9. After writing logs and debugging information to the files, move to step 4.

Building the new system requires performing a series of developments, including the following items:

- gathering data from different data sources: video cameras, microphones, sensors, smartphones;
- processing received data without delays or with minimal delays in the foggy environment regardless of the data type, complexity of the object structure and behaviour;
- defining spatiotemporal references for the models on the basis of the results of processing data received from sensors, recorded sound and video information;
- building the models of the subway stations as digital twins of the stations and the corresponding 2D and 3D models for the digital twin’s visualisation;
- designing a new architecture for the system, where the data is processed and stored in a storage centre in a fog computing environment;
- storage of the statistical information in a data lake (files) and in a DBMS database in the cloud;
- designing and developing three types of applications for end users: an application for smartphones, a website and a desktop application;
- design and development of an expert system web page with a separate administrator login/password that allows additional checks to be performed for cases of erroneous event detection;
- visualisation of events on the model in three-dimensional space for the technical personnel of the subway via their smartphones;
- providing operators with the possibilities to manage the situations that have emerged on subway stations and were detected based on registered events via smartphones or other types of devices.

The desktop program was specifically designed to grant operators access to the digital twins of subway stations, streamlining interactions between specialists and passengers. These digital twins are built based on individual plans of each subway station, enabling real-time updates through information received from sensors, audio, and video sources. The desktop application shares similarities with programs used in the production domain, where digital twins are employed to monitor and manage manufacturing processes of products and their components in real-time.

On the other hand, the website provides information about events and offers a subset of functions available in smartphone applications for iOS or Android. The visualization of a digital twin on the website is not intended due to the significant computational resources required for drawing 2D and 3D models without experiencing freezes. This limitation would hinder the usability of the website on devices with limited performance capabilities.

For smartphone applications, visualizing digital twins and providing real-time or near-real-time information about detected events are paramount. The part of the system located in the fog is deployed on a high-speed server, which was developed by Cisco CTO (https://www.cisco.com/) in 2011 called a fog computing environment, or is in the Microsoft Azure cloud (https://azure.microsoft.com/).

The authors confirm that all methods were carried out in accordance with relevant guidelines and regulations.

To estimate the efficiency of the developed system, a list of the efficiency indicators and criteria has been defined based on the brainstorming results^25,26,28^ (Table 1).

**Table 1:** Expected efficiency indicators and criteria.

No	Performance indicator	Expected result	Difference	Less, better, faster
1	Speed of application deployment in comparison with analogues	15 days	± 5	3 times
2	Scalability in comparison with analogues	yes		
3	The speed of building a digital twin in comparison with analogues	5 ms	± 2	2 times
4	Event detection accuracy with and without using an intelligent assistant	0.90	± 7	8%
5	The speed of staff response in comparison with analogues	10 min	± 8	30%
6	The latency when using foggy environments in comparison to using the cloud for data processing	15 ms	± 3	20 times
7	Energy savings when transferring computing to the foggy environment in comparison to computing on a PC	400 watts/hour	± 50	2 times

## Case study

### System development

Within the research, the following applications have been developed:

- an application for processing video and audio streams, data from sensors in the fog or cloud;
- three applications for monitoring, visualisation and control of events: an application for smartphones, a desktop Linux Gnome/KDE application, and a website with additional pages that allow control of errors using an expert system. Access to the additional pages is limited, and only users with administrator logins and passwords can go to these pages.

**Figure 7:**
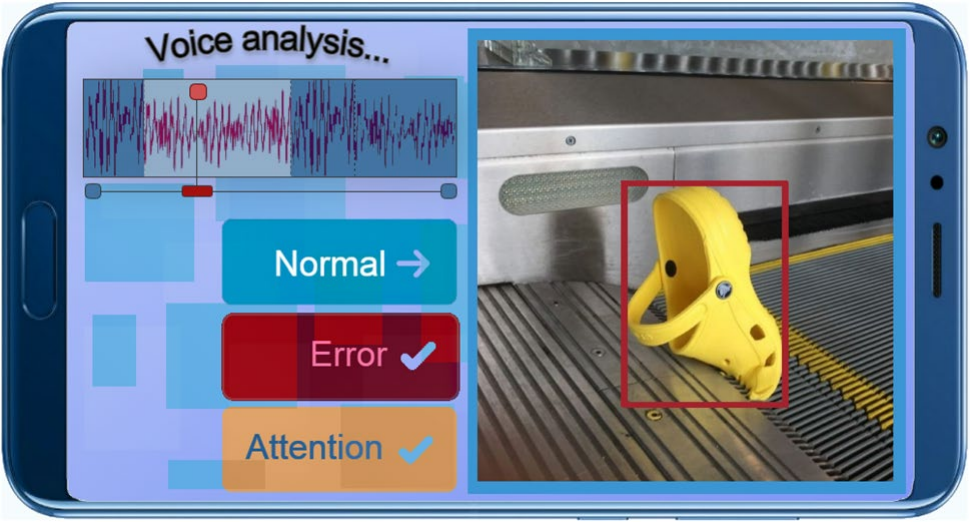
Intelligent assistant for the smartphones.

The application for the smartphone (Fig. 7) is endowed with the functions of an intelligent assistant and is not only intended for visual monitoring of the subway station but also for detecting events and identifying situations that are observed on the subway station at the defined moment of time based on gathered data. The arrangement of the elements on the main screen of the application is as follows. On the right part of the main screen the frame with the object that requires attention is placed; on the left side, there is a frame that provides the possibility for a specialist to listen to an excerpt from the audio stream on the smartphone; and in the centre part three buttons are located that allow identification of the situation based on the detected event. Three types of situations are possible: normal (everything is fine), error (an error that occurred in the process of event recognition using voice, sound and video) and attention (it is necessary to pay attention to the event; for further analysis, an expert system can be used). Information about the event is saved for logging, aggregation of statistics and subsequent analysis using an expert system. The expert system can be accessed through a website that can be opened on almost any device (macOS, PC, technological embedded computer and smartphone).

**Figure 8:**
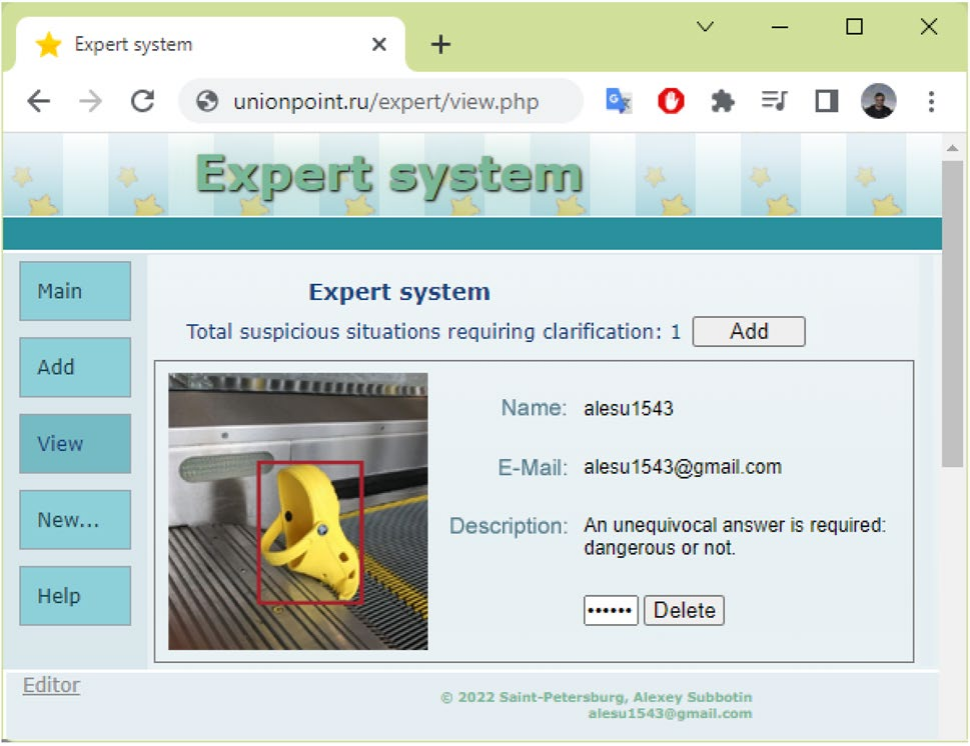
Expert system website.

The expert system site is hosted locally, but it can also be deployed on the internet (for example, http://unionpoint.ru). It is possible to launch the expert system only for users who have special logins/passwords (Fig. 8). With the help of the expert system, users can confirm or refute doubts about events by listening to sounds or viewing objects found in images, etc. On the screen, there is a button that allows adding the confirmed events to the list of detected events (“Add” button) and a button that allows marking the event as an error and deleting it from the list of the events (“Delete” button). When an event is deleted, the user enters his or her password, user name, and email address and provides a short description. On the left part of the screen, there is a menu with additional functions, through which, for example, it is possible to view a list of all situations.

**Figure 9:**
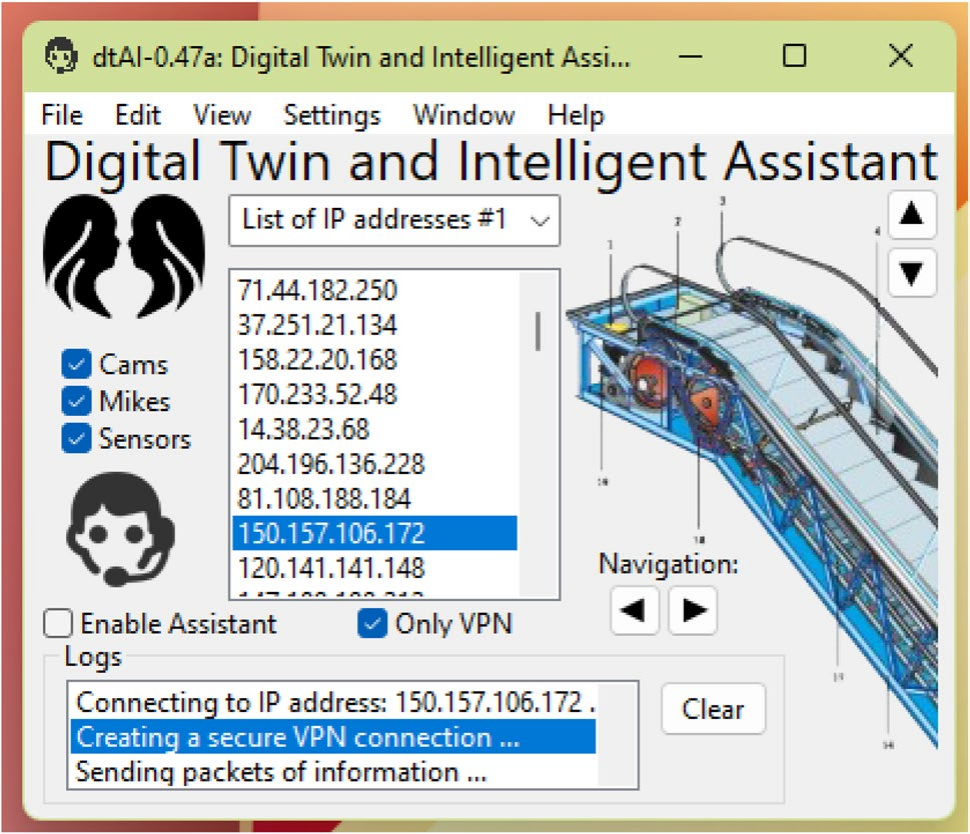
A desktop application of the system.

In the desktop application, it is possible to view the digital twin of the station that is presented based on the schemes of the real objects (Fig. 9). The application “dtAI-0.47a” shown in Fig. 9 was compiled and run under Windows 11, although it can also be run under Linux KDE/Gnome since it was developed in the Lazarus 2.0.10 cross-platform environment. On the right part of the main frame of the application, there is a scheme of the real object, and it is possible to move it and change its scale using a cursor arrow, which is more convenient than using the buttons that are placed at the top right and bottom left from the scheme. In the centre of the frame the list of IP addresses to connect to in a fog computing environment is enumerated. At the bottom part of the frame, there is the program log. On the left side there are options to select types of information for detecting events (video streams (Cams), sounds (Mikes), sensors (Sensors)) and the option that allows the use of intelligent assistant. If the option to use sound is enabled, then on the computer where the application is running the microphone must be turned on.

### Speed of the system deployment

After the system was developed, experiments to confirm its efficiency were conducted. The first indicator that was considered was the speed of deployment. Each of the analogue systems (Table 2) with which the proposed system is compared only had the module for intelligent video surveillance. In each system, only one object, in particular, a subway station, and no more than 20 video cameras have been considered. When estimating the indicator of the speed of deployment, the time of implementation, installation and deployment were considered. As a result of comparison of the indicator for Alexa, Alisa, etc., and the proposed system, it was found that the speed of deployment of the proposed system is on average 3.2 times (15.8/5 = 3.17) higher than that of its analogues. This is because we were using ready-made modules (programs, drivers for video cameras, etc.) with out of the box installation that were adapted based on the tasks that should be solved by the systems developed for the subway, in contrast to analogues that are not targeted on any particular domain. The time that was spent on the development of the proposed system includes the time spent on studying the documentation, revising the ready-made modules that needed adaptation and studying the subject domain with a subway specialist. The expectations were 15 ± 5 days, which are comparable with the results.

**Table 2:** Comparison of the speed of the developed system with analogous.

No	Speed of application deployment	Result
1	MDG Condor Project (1 module)	30–45 days
2	TsNII SET «Thames»	65–80 days
3	Blackbox based on Alisa	35–60 days
4	Google Assistant	30–40 days
5	Amazon Alexa	45–60 days
6	Microsoft Cortana	40–55 days
7	Proposed system “dtAI-0.47a”	15–20 days
	Faster on average:	3.2 times

### System scalability

An equally important indicator of the developed system that ensures the possibility of centralised and distributed deployment is its scalability (Table 3). Many assistants require additional development (Alisa, Alexa, Cortana and many others) to make them scalable, but the developed system is highly scalable because data processing is implemented in the fog or cloud, and the digital twin and intelligent assistant modules are separate programs that connect to servers after installation.

**Table 3:** Comparison of the scalability of the developed system with the analogues.

No	Scalability	Result
1	MDG Condor Project (1 module)	Yes
2	TsNII SET «Thames»	Yes
3	Blackbox based on Alisa	No
4	Google Assistant	No
5	Amazon Alexa	No
6	Microsoft Cortana	No
7	Proposed system “dtAI-0.47a”	Yes

### Speed of building a Digital Twin

The speed of building a graphical representation of a digital twin using “dtAI-0.47a” is much higher because two-dimensional space is used that mimics three-dimensional space. The graphics processing unit (GPU) is not used to redraw the twin. This approach has a number of limitations: it is impossible to rotate the model (clockwise and vice versa) along the X and Y axes at the same time and zoom in and out to see the tiny details of the object or its elements. However, at the same time, the speed of building these types of models is several times higher than using other approaches. The model is built in three-dimensional space only when the user of the digital twin explicitly requests this type of model. The constructed 3D model can be rotated using the mouse from the context menu, keys (Z + W) or by selecting the View item from the program menu. In most cases, using navigation buttons in a two-dimensional space (up-down, right-left) is sufficient. The speed of building a twin (Table 4) is on average 2.3 times higher than that in analogues, which is comparable to the expectations of 5 ± 2 ms.

**Table 4:** Comparison of the speed of building the digital twin in the developed system and in analogues.

No	The speed of building a digital twin	Result
1	Cooling system with motor (industrial fan on stand)	60–78 ms
2	Passenger elevator for business center	20–25 ms
3	Azure Manufacturing Digital Twins unveiled at Microsoft Build 2020	5–7 ms
4	IoT-based digital twins from Luxoft and DXC Technology Company	15–18 ms
5	Digital twin based on “dtAI-0.47a”	2–5 ms
	Faster on average:	2.3 times

### Event detection accuracy

The most important indicator for an intelligent surveillance system is the accuracy of event detection (Table 5). In the experiment, the accuracy is determined only by the usage of the intelligent assistant and not by the complexity of the algorithms of data processing that were applied. The accuracy increased by almost 10% (9.935), which is in line with expectations of 8%. The increase in accuracy could be much greater if more complex algorithms were used.

**Table 5:** Event detection accuracy with and without the use of the intelligent assistant.

No	Event detection accuracy	Before	Now	Result
1	Engine failure	0.83	0.95	12.6%
2	Insulation damage	0.87	0.97	10.3%
3	Breakage of the escalator tape	0.91	0.98	7.14%
4	Destruction of steps	0.89	0.96	7.3%
5	Poor lighting	0.82	0.94	12.8%
6	Flooding	0.86	0.95	9.47%
	Average:			9.935

### Reaction speed of staff and support services

The safety and sometimes the life of the passenger depends on the speed of the actions. In Table 6, the reaction rates of the personnel in four groups, electricians, hydraulics, mechanics, and security guards are presented. When evaluating the results, only the influence of intelligent assistant usage was taken into consideration, and the average reaction speed increased by more than 35%, although experts expected no more than 30%.

**Table 6:** The speed of staff response with and without the use of the intelligent assistant.

No	The speed of staff response	Before	Now	Result
1	Electricians	26 min	18 min	30.77%
2	Hydraulics	23 min	15 min	34.78%
3	Mechanics	12 min	7 min	41.67%
4	Guards	5 min	3 min	40%
	Average:			36.805

### Information processing speed

Using the fog computing environment reduced delays in data processing by 26.7 times in comparison to using the cloud (Table 7).

**Table 7:** Reduction of delays in data processing using the fog environment in comparison to using the cloud.

No	Latency reduction	Before	Now	Result
1	Mondays	391 ms	14 ms	27.93
2	Tuesdays, Wednesdays, Thursdays	354 ms	12 ms	29.5
3	Fridays	379 ms	13 ms	29.15
4	Saturdays	391 ms	17 ms	23
5	Sundays	383 ms	16 ms	23.93
	By average:			26.7

### Energy saving

Each year electricity consumption increases, the low cost of its production determines the well-being of the population, the state and all regions. However, the price per kWh may be unacceptably high if a large amount of electricity is consumed, and managers often make decisions to purchase equipment that reduces the amount of consumed energy for the profitability of the entire production. Saving energy is not only saving money but also a decisive factor in the acquisition, implementation and use of technologies. In Table 8, the amount of consumed energy by the proposed system and by the module from the STC “Condor” is compared. The results showed that the amount of saved energy increased up to 3.5 times, although the expectations were only 2 times. Energy specialists took into account the energy consumed in the Pyramida 2000 AWP program, ST2000-12 m, Pyramida IMS and SIKON S50 USPD.

**Table 8:** Electricity savings (kW/h) due to using the proposed system in comparison to the analogous system.

No	Energy saving (2022)		Before	Now	Result
1	March 14–April 20	37	11,410.8	2912.39	3.92
2	April 21–May 17	26	7169.76	1991.53	3.6
3	May 18–June 10	23	7198.3	2108.72	3.41
4	June 11–June 15	4	1221.12	346.56	3.52
5	June 16–June 21	5	1426.2	394.8	3.61
6	June 22–June 27	5	1443.4	382.6	3.77
7	June 28–July 1	3	858.96	245.52	3.5
	By average				3.618571

### Summary of the experiments and reproduction method

The results of all experiments are summarised in Table 9.

**Table 9:** The summarised results of the experimental research of the proposed system.

No	Performance indicator	Expected result	Received result
1	Speed of application deployment	3 times	3.2 times
2	Scalability in comparison with analogues	yes	yes
3	The speed of building a digital twin in comparison with analogues	2 times	2.3 times
4	Event detection accuracy with and without an intelligent assistant	8%	9.94%
5	The speed of staff response with and without an intelligent assistant	30%	36.8%
6	Reduced latency when using a fog environment in comparison to using the cloud for data processing	20 times	26.7 times
7	Energy savings when transferring computing to the fog environment in comparison to using a PC	2 times	3.6 times

To reproduce the experiment, it is necessary to create an intelligent assistant application, create a digital twin module, collect statistics provided in logs, open the collected data in Microsoft 365 and Office 2021, apply filtering, summation, finding the average and filling in the resulting tables. The following technical and program tools are needed: HPE Apollo 6500 server with Intel Xeon ProLiant XL270d Gen10 processor with NVLink 300 GB/s interprocessor interface (https://www.hpe.com/), smartphones: Xiaomi Poco M4 Pro 4G 6/128 GB and Samsung Galaxy A73 5G 8/128 GB for testing the assistant and a system unit for deploying and testing the digital twin with the parameters: Intel Core i5-11400F, 6 × 2.6 GHz, 16 GB DDR4, GeForce GTX 1660 SUPER, 512 GB SSD with preinstalled Ubuntu Linux 22.04 (https://ubuntu.com/) and MegaFon cloud (https://cloud.megafon.ru/) to test the transfer of computing from the cloud to the fog environment.

## Conclusion

This article delves into the development of an intelligent assistant tailored for subway operations, leveraging a multilevel spatiotemporal model. Through an in-depth review of analogous systems and exploration of similar applications in other domains, existing challenges are highlighted, leading to the proposal of a novel system grounded in intelligent assistants. The newly proposed system showcases remarkable advancements, offering threefold improvement in deployment speed, twofold acceleration in model construction, nearly 10% higher accuracy in event determination, and an impressive 37% increase in staff response speed. These achievements can be attributed to the elevated intelligence level of the assistants employed in the system. The proposed approach has shown its effectiveness through the example of the digital twin of a subway station, but it can also be applied to other areas, although this can require further development of the proposed assistance.

## Data Availability

All the necessary data to repeat the experiments is here: https://github.com/alex1543/multilevel.
